# Habitual intake of dietary L-arginine in relation to risk of type 2 diabetes: a prospective study

**DOI:** 10.1186/s12902-021-00774-x

**Published:** 2021-05-31

**Authors:** Parvin Mirmiran, Zahra Bahadoran, Zahra Gaeini, Fereidoun Azizi

**Affiliations:** 1 Nutrition and Endocrine Research Center, Research Institute for Endocrine Sciences, Shahid Beheshti University of Medical Sciences, P.O. Box: 19395-4763, No. 24, Sahid-Erabi St, Yemen St, Chamran Exp, Tehran, Iran; 2 Endocrine Research Center, Research Institute for Endocrine Sciences, Shahid Beheshti University of Medical Sciences, Tehran, Iran

**Keywords:** L-arginine, Dietary protein, Type 2 diabetes

## Abstract

**Background:**

There are insufficient data in case of the potential association of habitual dietary L-arginine and the risk of type 2 diabetes mellitus (T2DM) incidence. Here we aimed to examine the potential effect of dietary L-arginine on the T2DM incidence.

**Methods:**

For this cohort study, 2139 T2DM-free adults from the participations of Tehran Lipid and Glucose Study (TLGS) were recruited. Follow up period was approximately 5.8 years. Daily intakes of protein and L-arginine were estimated using a validated food frequency questionnaire with 168 food item. Hazard Ratios (HRs) and 95% confidence intervals (CIs), adjusted for sex, age, smoking, diabetes risk score, physical activity levels, and total energy intakes as well as carbohydrate, fiber, fats and lysine, were calculated for L-arginine as both absolute intake and its ratio from total protein.

**Results:**

Mean (±SD) age of the participants was 38.9 (±12.6) years and 54.6% were women. Mean (±SD) intake of dietary protein and L-arginine was 77.2 (±22.4) and 4.05 (±1.50) g/d, respectively. An increased risk of T2DM (HR = 2.71, 95% CI = 1.20–6.09) was observed among participants with higher intakes of L-arginine (median intake of > 5.4 vs. 2.69 g/d). Total protein intake and the ratio of L-arginine to total protein intakes were not related to incidence of T2DM in both crude and adjusted models.

**Conclusion:**

We found that higher dietary L-arginine levels may increase risk of T2DM and it may have an independent role in T2DM development.

## Background

L-arginine is a conditionally essential amino acid that involved in the synthesis of proteins, creatine, polyamines, agmatine, urea, and metabolism of proline and glutamate in the body [1, 2]. The relative amount of L-arginine in different dietary proteins is in a range of 3–15% [3]; usual daily intakes of the Arg has been estimated to be 4–6 g per day in healthy adults, which provide about 20% of plasma L-arginine flux [4]. L-arginine has recently received more interest as a nitric oxide (NO) precursor, a property has led to the widespread use of L-arginine as a complementary therapy in various NO-disrupted conditions [5, 6].

Short-term beneficial properties of L-arginine supplementation in some pathologic conditions including hypertension, hypertensive renal disease and cardiovascular disease have been investigated [7–9]. Several studies suggested that L-arginine may be involved in multiple NO-dependent pathways that affect the glucose and insulin homeostasis [10, 11]. Beyond its effects through NO metabolism, L-arginine has direct effect in stimulation of insulin secretion in diabetic rats [12], and it was suggested that L-arginine stimulated glucose-induced insulin secretion in mouse by membrane depolarization, independently of NO, in another observational study [13]. However, due to lack of efficacy and safety of L-arginine supplementation in long-term period (e.g. increased risk of mortality rate and myocardial infarction following 6 months of 9 g/d L-arginine supplementation) [14, 15], along with undesired effects of L-arginine (e.g. infusion of arginase activity and increased urea levels), the dominant paradigm about beneficial effects of L-arginine is under debate [16, 17]. In our previous studies we showed that dietary intakes of L-arginine were positively related to NO metabolites levels in serum [18], and increased chance of chronic kidney disease incidence [19], metabolic syndrome [20], and coronary heart disease incidence [21].

Although there are several animal studies investigated the effects of L-arginine supplementation on glucose and insulin homeostasis, long-term effects of L-arginine intake from usual diet in human is unclear. To the best of our knowledge, there is limited data in case of the association of habitual L-arginine intakes from diet and the risk of T2DM, therefore, in this study we aimed to evaluate the possible association of dietary L-arginine, as both absolute intake and its ratio from total protein intake, with the incidence of T2DM in a population-based study.

## Methods

### Study population

This prospective cohort study was conducted using data collected from the Tehran Lipid and Glucose Study (TLGS). TLGS is an ongoing community-based cohort study, in a sample in the district 13 of Tehran, Iran, aimed to investigate and prevent non-communicable diseases [22]. We recruited 3462 men and women from the participants of the third phase of the TLGS, who had completed dietary and demographic data.

**Fig. 1:**
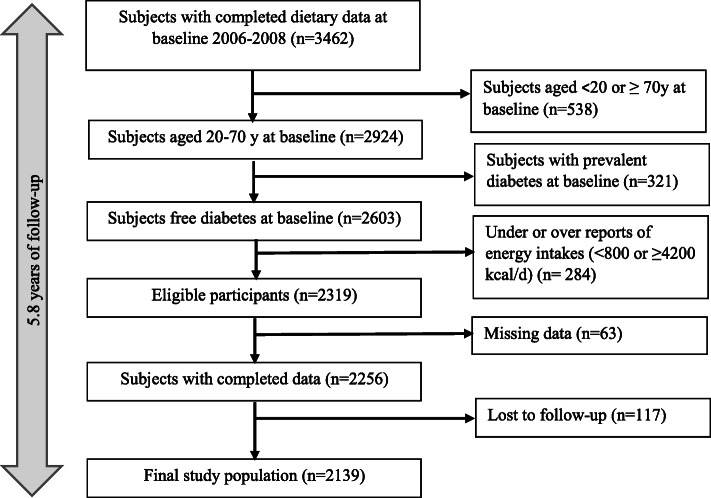
The flowchart of the study

Finally, after exclusion of subjects with T2DM diagnosis at baseline (*n* = 321), participants who had missing data of anthropometrics, biochemical values and physical activity (*n* = 63), and those with under- or over-report of total energy intakes (< 800 kcal/d or > 4200 kcal/d) (*n* = 284), 2256 adults were remained and followed up to the fourth and fifth TLGS examinations, ~ 3 years apart. Mean period of follow-up was 5.8 years. Final analyses were conducted on 2139 adults (971 men, 1168 women), after exclusion of participants who had no follow-up after the baseline examination (*n* = 117). The flow chart of selection of study population is shown in Fig. 1. The participants with lost to follow-up and missing data were considered as non-responders; accordingly, the response rate of the study was 92.2%.

The study protocol was approved by the ethics research council of the Research Institute for Endocrine Sciences, Shahid Beheshti University of Medical Sciences. Moreover, written informed consents were obtained from all participants.

### Demographic and anthropometric measures

Demographic data were conducted by the trained interviewers, using standard questionnaires. Measurements of demographic variables in TLGS have been reported elsewhere in details [22]. Body weight and height of participants were measured using digital scales and a tape meter, to the nearest 100 g and 0.5 cm, respectively. Subjects were in a standing position, without shoes and minimally clothed for anthropometric measurements. Body mass index (BMI) was calculated as weight (kg) divided by square of the height (m^2^). Furthermore, waist circumference (WC) was measured to the nearest 0.1 cm, over light clothing, between the lower border of the ribs and the iliac crest at the widest portion. WC was measured using a soft measuring tape, with no pressure to the body. Waist to height ratio (WHtR) was calculated as WC divided by height (cm).

To assess both systolic (SBP) and diastolic blood pressure (DBP) of participants, two measurements of blood pressure were taken, with at least a 30-s interval between two measurements. There was a 15-min rest before blood pressure measurements, and subjects were in the sitting position. For this purpose, we used a standardized mercury sphygmomanometer calibrated by the Iranian Institute of Standard and Industrial Research. Final blood pressure of participants was considered as the mean of the two measurements.

To assess physical activity level of participants, the frequency and time spent on light, moderate, high and very high intensity activities, according to the list for common activities of daily life were asked over the past year. We used metabolic equivalent hours per week (METs h/week) to express physical activity levels.

### Biochemical measures

Participants had a 12–14 h of overnight fasting before biochemical measurements. Blood samples were drawn between 7:00 and 9:00 AM. Fasting serum glucose (FSG) and serum triglyceride (TG) were assayed using glucose oxidase and glycerol phosphate oxidase, respectively. The method used to measure FSG and TG was enzymatic colorimetric. High-density lipoprotein cholesterol (HDL-C) was measured after precipitation of the apolipoprotein B containing lipoproteins with phosphotungstic acid. Analyses were performed using Pars Azmoon kits (Pars Azmoon Inc., Tehran, Iran) and a Selectra 2 auto-analyzer (Vital Scientific, Spankeren, Netherlands). Both inter- and intra-assay coefficients of variation of all assays were < 5%.

### Dietary assessment

Typical food intakes were assessed using a validated 168-item FFQ. The validity and reliability of the questionnaire have been previously evaluated [23]. The intake frequencies for each food item during the past year were asked on a daily, weekly, or monthly basis. Then we converted the reported portion sizes in household measures to grams [24]. To analyze the energy and nutrient contents of foods and beverages, we used the US Department of Agriculture Food Composition Table, because the Iranian Food Composition Table is incomplete, and has limited data [25]. To obtain dietary total intake of L-arginine, the L-arginine content of food items (mg/100 g of foods) were multiplied by the amount of daily intake of food items, then the obtained values were summed up [18].

### Definition of terms

A T2DM patient considered as a subject who met at least one of the following criteria: (1) using anti-diabetic drugs, (2) fasting serum glucose (FSG) ≥126 mg/dL, (3) 2-h post challenging glucose (2-hPCG) ≥200 mg/dL [26]. If they had at least one parent or sibling with T2DM, a positive family history of diabetes was considered for them. To calculate the diabetes risk score (DRS), we summed up the points considered for each following items: SBP (mm Hg) < 120 (0 point), 120 < SBP < 140 (3 point), SBP ≥ 140 (7 point); waist to height ratio (WHtR): < 0.54 (0 point), 0.54–0.59 (6 point), ≥0.59 (11 point); family history of diabetes (5 point); FSG (mmol/L): < 5 (0 point), 50–5.5 (12 point), 5.6–6.9 (33 point); TG/HDL-C: < 3.5 (0 point), ≥3.5 (3 point) [27].

### Statistical analysis

Mean and standard deviation (SD) values, and the frequency (%) of baseline characteristics were compared across tertiles of L-arginine intakes, using independent analysis of variance or chi square test, respectively. Hazard ratios (HRs) and 95% confidence intervals (CIs) for the association between L-arginine intakes and L-arginine to protein ratio in relation to incidence of T2DM were estimated using Cox proportional hazards regression models with person-year as the underlying time metric. Cox models were adjusted for sex, age, smoking, diabetes risk score, physical activity levels, and total energy intakes as well as dietary carbohydrate, fiber, fats and lysine.

The middle-time between the date of the first diagnosis of T2DM, and the most recent follow-up visit preceding the diagnosis were considered as the event date for T2DM cases. The difference between the calculated middle-time date and the date at which the subjects entered the study was considered as the follow-up time. In case of the censored and lost to follow-up subjects, the survival time was the interval between the first and the last observation dates. Follow-up duration and person-years were calculated using the measured survival time.

All analyses were performed using IBM SPSS for Windows version 20, with a two-tailed *P* value< 0.05 being considered significant.

## Results

Mean (SD) age of the participants was 38.9 (12.6) years and 45.4% were men. Mean (SD) BMI was 26.9 (4.7) kg/m^2^ at baseline. A total of 143 cases of T2DM were identified over a median 5.8 years of follow-up. Mean (SD) intake of dietary protein and L-arginine was 77.2 (22.4) and 4.05 (1.50) g/d, respectively. There were no significant differences between dietary intakes of L-arginine, total protein and L-arginine/protein ratio in subjects with or without T2DM (4.05 ± 1.5 g/d vs. 4.05 ± 1.51 g/d, 75.91 ± 25.95 g/d vs. 77.39 ± 27.56 g/d, 0.053 ± 0.006 vs. 0.053 ± 0.006, respectively).

Baseline characteristics of participants are shown in Table 1. Participants in the highest compared to the lowest intake of L-arginine were more likely to be younger (38.5 vs. 40.6 years, *P* = 0.006), and had higher physical activity (36.8 vs. 30.4 MET-h/week, *P* = 0.08). There was no statistically significant difference in anthropometric and biochemical values as well as diabetes risk score across dietary intakes of L-arginine. Dietary intake of carbohydrate, fiber and protein was significantly increased with higher intakes of L-arginine, whereas intakes of total fat decreased (*P* for all < 0.01).

**Table 1:** Characteristics of the study population across tertiles of dietary L-arginine intakes

	*Dietary L-arginine*			
	*Tertile 1*	*Tertile 2*	*Tertile 3*	P^*^
L-arginine *(g/d)*				
Range	< 3.31	3.31–4.45	> 4.45	
Median	2.69	3.77	5.40	
Age *(y)*	40.6 ± 12.8	39.4 ± 12.3	38.5 ± 12.6	0.006
Men *(%)*	35.1	45.6	55.5	0.001
Current smoker *(%)*	10.2	14.2	12.5	0.011
Physical activity *(MET-h/week)*	30.4 ± 45.0	33.7 ± 52.0	36.8 ± 57.0	0.086
BMI *(kg/m*^*2*^*)*	27.0 ± 4.8	26.8 ± 4.7	27.1 ± 4.7	0.448
FSG *(mmol/L)*	4.81 ± 0.47	4.80 ± 0.44	4.84 ± 0.47	0.186
2-hPCG *(mmol/L)*	5.36 ± 1.41	5.21 ± 1.31	5.28 ± 1.37	0.098
TG to HDL-C ratio	1.66 ± 0.2	1.65 ± 0.2	1.68 ± 0.2	0.680
Diabetes risk score	9.3 ± 10.0	9.1 ± 10.0	9.7 ± 10.1	0.564
Dietary intakes				
Carbohydrate *(% of energy)*	57.0 ± 7.5	57.7 ± 6.5	58.1 ± 7.4	0.030
Fat *(% of energy)*	32.4 ± 7.5	31.3 ± 6.6	29.9 ± 6.6	0.001
Protein *(% of energy)*	12.9 ± 2.2	13.6 ± 3.1	14.5 ± 2.6	0.001
Total fiber *(g/1000 kcal)*	15.9 ± 6.9	16.5 ± 3.4	17.0 ± 6.8	0.001
L-arginine to protein ratio	0.051 ± 0.01	0.052 ± 0.01	0.054 ± 0.01	0.001

Data are mean ± SD unless stated otherwise (Analysis of variance and chi-square test was used for continuous and dichotomous variables, respectively)

Association between L-arginine intakes as well as dietary protein and L-arginine to protein ratio with the incidence of T2DM after a 5.8 y of follow-up are shown in Table 2. In the crude model, there was no statistically significant association between intakes of L-arginine and risk of T2DM (HR = 0.83, 95% CI = 0.55–1.26, and HR = 1.06, 95% CI = 0.71–1.56, in the second and third tertile, respectively). After adjustment of diabetes risk score, physical activity, sex, age, smoking, total energy intakes, and carbohydrates, fiber, fats, lysine and total protein intakes, the risk of T2DM significantly increased in the highest tertile compared to the lowest tertile categories of L-arginine intakes (HR = 2.71, 95% CI = 1.20–6.09). Total protein and the ratio of L-arginine to total protein intake were not significantly associated with risk of T2DM (HR = 1.89, 95% CI = 0.99–3.60), in both crude and adjusted models. It is notable that there was no sex interaction between L-arginine and T2DM in our study**.**

**Table 2:** The risk of type 2 diabetes across tertile categories of dietary L-arginine, dietary protein and L-arginine to protein ratio

	Hazard Ratio (95% CI)			
	*T*_*1*_	*T*_*2*_	*T*_*3*_	P for trend^*^
L-arginine				
*Crude*	*Ref.*	0.83 (0.55–1.26)	1.06 (0.71–1.56)	0.430
*Model 1*	*Ref.*	1.00 (0.65–1.54)	0.95 (0.62–1.45)	0.816
*Model 2*	*Ref.*	1.44 (0.84–2.46)	2.72 (1.21–6.08)	0.019
*Model 3*	*Ref.*	1.44 (0.84–2.47)	2.71 (1.20–6.09)	0.020
Total protein				
*Crude*	*Ref.*	0.90 (0.60–1.35)	0.94 (0.63–1.40)	0.494
*Model 1*	*Ref.*	1.33 (0.83–2.12)	1.46 (0.94–2.27)	0.099
*Model 2*	*Ref.*	1.42 (0.84–2.39)	1.89 (0.99–3.60)	0.052
L-arginine to protein ratio				
*Crude*	*Ref.*	1.07 (0.70–1.64)	1.45 (0.97–2.16)	0.145
*Model 1*	*Ref.*	1.02 (0.64–1.61)	1.28 (0.84–1.95)	0.242
*Model 2*	*Ref.*	0.93 (0.58–1.50)	1.22 (0.78–1.90)	0.371

Hazard ratio and 95% confidence interval; Cox regression models were used

Model 1: Adjusted for sex, age, smoking, diabetes risk score and physical activity

Model 2: additional adjustment for total energy intakes, dietary carbohydrate, fiber, fat and lysine

Model 3: additional adjustment for total protein intake

*P* for trend test was performed by considering each ordinal score variable as a continuous variable in the model

## Discussion

In the current prospective population-based study, we showed a potential adverse effect of high L-arginine intakes (more than 5.4 g/d) from habitual diet in relation to risk of T2DM, during 5.8 years of follow-up. Higher intakes of L-arginine however were not a potential risk for development of T2DM when it was considered in the context of total protein intake, as L-arginine-to-protein ratio.

Mean dietary intake of L-arginine was 4.0 ± 1.5 g/d in our population, with major dietary sources of grains and meats. Although there is no recommended dietary allowance for L-arginine [4], mean dietary intake of L-arginine has been reported 4–6 g/d in different population [28, 29]. Different dietary sources have 3–15% of L-arginine, with highest amount of L-arginine for soy protein, peanuts, walnuts, and fish; whereas cereal proteins are poor sources of L-arginine (3–4% of total amino acids) [30]. Different dietary patterns among population are responsible for a wide range of dietary intakes and plasma concentrations of L-arginine worldwide [21, 30].

Limited data are available regarding the possible association of dietary L-arginine and the cardio-metabolic outcomes. Low intakes of L-arginine (below the median range of 3.8 g/d) has been reported to be associated with the higher C reactive protein (CRP) levels, while the highest level of L-arginine intake (> 7.5 g/d) increased risk of high-CRP levels by 30% [29]. Also, higher dietary intakes of L-arginine was associated with significantly higher risk of coronary heart disease (CHD) incidence (relative risk = 1.87, 95% CI = 1.06–3.29 for intakes of L-arginine in a range of 3.86–4.65) [31]. L-arginine intake from animal sources was also related to higher diastolic blood pressure and increased risk of CHD events (hazard ratio = 1.90, 95% CI = 1.03–3.58) [21]. In our previous study, participants with higher intakes of L-arginine from animal sources had significantly higher risk for metabolic syndrome incidence (odd ratios =1.49, 95 95% CI = 1.02–2.18) [20]. Similarly, subjects with the highest amount of dietary animal-derived L-arginine, compared to whom with the lowest intakes (2.57 vs. 1.05 g/d) had significantly increased risk of chronic kidney disease (relative risk = 1.54; 95% CI = 1.06–2.14); L-arginine intakes from animal sources were also associated with decreased estimated glomerular filtration rate and creatinine clearance rate [19].

Patients with T2DM had higher plasma L-arginine concentration (median = 9.74, inter-quartile range = 5.33–16.61 vs. median = 4.47, inter-quartile range = 3.07–6.70); L-arginine concentration was positively associated with T2DM (odds ratio = 1.20, 1.17–1.23) [32]. A 10-year follow-up study also indicated that high levels of L-arginine significantly increased risk of T2DM by 21% (hazard ratio = 1.21, 95% CI = 1.07–1.37) [33]. A meta-analysis demonstrated that plasma L-arginine concentration is positively related to elevated risk of T2DM (pooled estimated relative risk = 1.19, 95% CI = 1.14–1.25) [34].

The biological plausible mechanisms linking L-arginine intake to risk of T2DM are not well documented. One possible explanation may be related to induction of arginase activity by long-term high intakes of L-arginine [2, 17], that has been suggested may contribute to the development of T2DM and insulin resistance [35]. The elevated levels of L-arginine is suggested to increase urea synthesis, because L-arginine induces N-acetylglutamate synthase which further activates carbamoyl phosphate synthetase-I (CPS-I) to start urea cycle [32]. L-arginine may also decrease cellular uptake of citrulline [36], interrupt recycling of L-arginine form citrulline [17], and suppress endothelial NO synthase (eNOS) expression and activity [37], resulting decreased eNOS-derived NO and development of insulin resistance. On the other hand, high-L-arginine intakes is suggested to be related with pathologic levels of NO metabolites [38–40], possibly produced by inducible NO synthase (iNOS); higher intakes of L-arginine were associated with higher serum NO metabolites [18, 20], an independent risk factor of cardio-metabolic diseases [38, 40, 41].

Further prospective studies are needed to more fully determine the possible underlying mechanisms by which high intakes of L-arginine increased risk of T2DM.

The population-based prospective setting of the current study, and use of a validated FFQ to assess regular dietary intake are the main strength points of the study. Due to low number of outcomes (*n* = 143), we used the DRS in multivariate models, which allowed us to not adding many variables that would lead to instability of our models, and accounts for major T2DM confounders . However it had some limitations. First, we had not data on serum levels of L-arginine to consider in our analysis; however, an acceptable correlation was observed between dietary L-arginine intakes and serum L-arginine. Some inherent limitation points of observational studies including potential selection bias, information bias in measuring L-arginine intakes as study exposure, and non-differential misclassification of the exposure also should be considered. Furthermore, there are other potential confounding variables, including some dietary factors, which could affect the association of L-arginine and T2DM and were not included in our adjusted models; since L-arginine is derived from different food sources, other dietary factors occurring in the same foods could also have affected the findings. Other potential limitation of the present study is the possible changes of dietary patterns during the follow-up period, since we assessed the dietary information only at baseline examinations; however previous observations in our population indicates an acceptable stability of major dietary patterns over the time.

## Conclusion

Our findings from this prospective study indicated that higher amount of dietary L-arginine may be potential risk factor for development of T2DM. Considering the increasing interest to ingestion of L-arginine as a popular dietary supplement, and also the limited data in case of the potential association between dietary L-arginine and cardio-metabolic outcomes especially T2DM, further cohort studies are required to clarify the possible association.

## Data Availability

The datasets used and/or analyzed during the current study available from the corresponding author on reasonable request.

## Abbreviations

BMI: Body Mass Index
CI: Confidence Intervals
CHD: Coronary Heart Disease
C-RP: C - reactive protein
DBP: Diastolic Blood Pressure
DRS: Diabetes Risk Score
FSG: Fasting Serum Glucose
FFQ: Food Frequency Questionnaire
HDL-C: High-Density Lipoprotein Cholesterol
HR: Hazard Ratios
LDL-C: Low-Density Lipoprotein Cholesterol
MET: Metabolic Equivalent
NO: Nitric Oxide
SBP: Systolic Blood Pressure
TLGS: Tehran Lipid and Glucose Study
T2D: Type 2 Diabetes
TG: Triglyceride
WC: Waist Circumference
WHtR: Waist to Height Ratio
2-hPCG: 2-h Post Challenging Glucose
